# Longitudinal predictor analysis of pain in breast cancer survivors on aromatase inhibitors—the PAC-WOMAN trial

**DOI:** 10.3389/fpain.2026.1700365

**Published:** 2026-05-14

**Authors:** Inês Nobre, Marlene N. Silva, Sofia Franco, Bruno Rodrigues, Pedro F. Silva, Pedro J. Teixeira, Eliana V. Carraça

**Affiliations:** 1 CIPER, Faculdade de Motricidade Humana, Universidade de Lisboa, Cruz Quebrada, Portugal; 2 Centro de Investigação em Educação Física, Desporto, Exercício e Saúde (CIDEFES) E CIFID2D, Universidade Lusófona, Lisboa, Portugal; 3 CISUC, Universidade de Coimbra, Polo II, Coimbra, Portugal

**Keywords:** aromatase inhibitors, breast cancer pain, breast cancer survivors, hormonal therapy, pain predictors, physical activity interventions

## Abstract

**Objective:**

Breast cancer survivors undergoing hormonal therapy with aromatase inhibitors face a significantly increased risk of developing musculoskeletal pain, leading to treatment discontinuation rates of 13% to 22%. Recent literature has focused on identifying and understanding possible risk factors for pain development in breast cancer survivors, not only for pain prevention but also for guiding treatment strategies. This study aimed to investigate predictors of baseline, 4-month, and change scores of pain intensity in breast cancer survivors on aromatase inhibitors.

**Methods:**

Multiple regression analyses using intent-to-treat data (*N* = 110; mean age 56.2 ± 7.7 years) were performed to identify possible predictors applying a biopsychosocial approach with five domains: sociodemographic characteristics, treatment-related factors, health-related factors, physical function factors, and group allocation. The Brief Pain Inventory was used to assess participants' pain intensity.

**Results:**

Having a partner (95% CI: 0.220, 1.868, *p* = 0.014), lower sleep quality (95% CI: −1.694, −0.615, *p* < 0.000), and additional time on the Timed-up-and-go test (95% CI: 0.002, 1.185, *p* = 0.049) were significant predictors of higher baseline pain intensity. Significant predictors of higher 4-month pain included lower sleep quality (95% CI: −1.095, −0.054, *p* = 0.031), lower chest press strength (95% CI: −0.231, −0.032, *p* = 0.010) and being in the brief physical activity counseling group (95% CI: 0.324, 2.155, *p* = 0.008) relative to the exercise group. In the change scores model, compared to the exercise group, being in the brief physical activity counseling group (95% CI: 0.183, 1.068, *p* = 0.006), and waitlist control group (95% CI: 0.054, 0.947, *p* = 0.029) significantly predicted an increase in pain intensity over time. Conversely, having a partner (95% CI: −0.825, −0.057, *p* = 0.025) significantly predicted a reduction in pain intensity.

**Conclusions:**

These findings highlight the complex and multifaceted nature of pain in this population and the need for longitudinal studies with diverse populations, as well as advanced methodologies to capture the dynamic nature of pain and refine intervention strategies.

## 1 Introduction

Breast cancer is the most diagnosed cancer in women, with an estimated 2.3 million new cases in 2022, accounting for 23.8% of all cancer cases worldwide (1). Due to earlier detection and improvements in treatment, breast cancer survival rates have increased substantially over time (2). The 5-year relative survival rates at an early or localized stage are, on average, 96% and 91% in the EU and the US, respectively (3, 4).

While survivorship is increasing, new challenges arise, impacting the quality of life of breast cancer survivors (BCS) (2, 3). Pain, related to both surgical and non-surgical interventions, affects nearly 30% of BCS (5), leading to negative impacts on physical and mental health, social functioning, and quality of life (6, 7). Pain is also associated with an increased risk of unemployment and with greater healthcare costs (8, 9).

BCS undergoing hormonal therapy with aromatase inhibitors (AIs) face a significantly increased risk of developing musculoskeletal pain, including myalgia and arthralgia. Studies report a 36% to 80% pain prevalence among women receiving AI therapy, leading to treatment discontinuation rates of 13% to 22% (10). Discontinuing AI therapy can compromise treatment efficacy and potentially increase the risk of cancer recurrence (11).

Recent literature has focused on identifying and understanding possible risk factors for pain development in BCS, not only for pain prevention but also for guiding treatment strategies (12, 13). Given the multidimensional nature of the survivorship experience, the biopsychosocial model of cancer pain (14) has emerged as a primary framework. This model states that pain is not merely a physiological signal of tissue damage but an emergent experience resulting from the reciprocal influence of biological, psychological, and social factors. Consequently, a growing body of research has adopted this approach to explore BCS pain predictors, considering various domains, including socio-demographic characteristics, treatment-related factors, and health-related variables (13, 15, 16).

Evidence suggests pain is more prevalent among BCS with low educational attainment, low income, and younger age (17, 18). Treatment-related aspects, including disease stage, breast cancer surgery, chemotherapy, and radiotherapy, are positively associated with BCS pain (7, 19). Health factors such as obesity, diabetes, smoking, and depression are significantly associated with higher odds of pain (18, 20, 21). On the contrary, physical activity (PA) and non-sedentary activity are associated with significantly lower pain frequency (18, 22).

PA's role in enhancing BCS quality of life and mitigating short- and long-term adverse effects related to cancer treatments is well-established (23–25). Physically active BCS have significant improvements in their physical function, strength, cardiorespiratory fitness, flexibility, and body composition, and experience decreased fatigue, depression, anxiety, stress, and sleep disorders (26–28). Currently, PA guidelines for cancer survivors target cancer-related health outcomes and recommend regular participation in aerobic exercise, resistance training, or a combination of both modalities (29).

Furthermore, a growing body of evidence supports the impact of PA interventions on BCS pain management (22, 30–32). However, while supportive, the literature regarding PA's impact on pain management for BCS receiving AI therapy remains limited (33). Further exploration is needed to elucidate potential mechanisms and guide more effective treatment strategies.

Most studies in BCS that investigated predictors of pain have relied on cross-sectional data (19). However, through a prospective study, Johannsen et al. (34) demonstrated that the predictive strength of factors associated with persistent pain might change over time. Additionally, there is a lack of research exploring how pain predictors influence pain trajectories during PA interventions. Studying prospective predictors of pain changes in PA interventions might help identify factors that enhance or undermine pain persistence and improve the tailoring of PA interventions to BCS needs.

Therefore, the present study, an ancillary analysis of the PAC-WOMAN trial, a 3-arm pragmatic randomized controlled trial for BCS undergoing AI therapy (35), investigated baseline predictors of pain intensity before and after a physical activity intervention, organized into five domains: sociodemographic characteristics, treatment-related factors, health-related factors, physical function factors, and group allocation. Specifically, this study (i) analyzed these factors as predictors of baseline, 4-month, and change scores of pain intensity; and (ii) compared potential predictors amongst pain intensity tertiles.

## 2 Materials and methods

### 2.1 Study design and setting

PAC-WOMAN is a three-arm pragmatic randomized controlled trial (RCT) designed to investigate the long-term effectiveness of different interventions in promoting active lifestyles and improving the quality of life in BCS receiving AIs. The trial has a 4-month intervention period with a 12-month follow-up and compares a brief PA counseling and supervised structured exercise with a waitlist control group.

### 2.2 Participants and recruitment

Participant recruitment was achieved mainly via medical referrals from several main hospitals with oncology centers. Informative brochures for physicians and patients were produced and delivered in recruiting hospitals.

The inclusion criteria for this study were: 1) be post-menopausal women, below 70 years old; 2) histologically confirmed hormone-receptor-positive breast cancer (stage I, II, III); 3) have initiated AI hormonal therapy following the primary treatment (surgery, radiotherapy, chemotherapy, etc.), at least 1 month before being enrolled; and 4) be able to perform light-to-moderate physical activities (ECOG 0-1). Participants were excluded if they had one or more of the following conditions: 1) stage IV cancer or synchronous tumors; 2) uncontrolled hypertension, cardiac or pulmonary disease; 3) contraindications to exercise training according to the assistant doctor; 4) inability to provide informed consent; or 5) expected inability to fulfill the proposed program schedule.

All participants provided written informed consent before enrollment. Following baseline assessments, participants were randomly allocated to one of three groups: brief PA counseling, supervised structured exercise, or waitlist control.

### 2.3 Measures

Participants were assessed in small groups at baseline and 4 months (intervention's end), in standardized conditions, in the exercise setting, and in a calm, comfortable environment.

#### 2.3.1 Pain

Brief Pain Inventory (BPI) (36) was used to assess participants' pain intensity/severity on average. The BPI is scored on a 10-point Likert-type scale from 0 (“No pain”) to 10 (“Pain as bad as you can imagine”). Pain intensity on average was reported at baseline and 4-month assessments, and pain change scores were calculated using the residualized change scores.

#### 2.3.2 Baseline predictors

To capture the multidimensional nature of pain experience in BCS undergoing AI therapy, predictor selection was guided by the biopsychosocial model of cancer pain (14). In line with this, variables were systematically categorized into five domains, comprising sociodemographic, treatment-related, health-related, physical function, and intervention groups, to identify baseline predictors of pain intensity at baseline, 4 months, and change scores.

##### 2.3.2.1 Sociodemographic

To account for the social and economic context, sociodemographic variables included participants' reported age (years at questionnaire completion), relationship status (0: single; 1: having a partner), education level (5-point Likert-type scale from 1 “elementary school” to 5 “master's degree or higher”), and financial situation perception (3-point Likert-type scale from 1 “difficult” to 3 “comfortable”).

##### 2.3.2.2 Treatment-related

Biological burden and treatment history were assessed to account for potential physiological sensitization. Variables included: breast cancer stage (from I to III), time since diagnosis, time in hormonal therapy with AIs (months at questionnaire completion), and participants’ treatment types (mastectomy, lumpectomy, axillary lymph node dissection, expander placement, chemotherapy, and radiotherapy; all coded as 0: no; 1: yes).

##### 2.3.2.3 Health-related

This domain focused on psychological and systemic factors known to modulate pain thresholds. We included variables such as chronic diseases (0: no chronic diseases; 1: having chronic diseases), depression score (using the Hospital Anxiety and Depression Scale), sleep time (minutes, measured with Actigraph GT9X wrist accelerometer), sleep quality (assessed with the Pittsburgh Sleep Quality Index), waist circumference (cm, measured according to the NIH protocol with SECA, Germany), and fat mass percentage (bioelectrical impedance with Impedimed, Australia).

##### 2.3.2.4 Physical function

To investigate the relationship between musculoskeletal conditioning and pain, objective physical function measures were used: handgrip strength (kg) (37), Sit-to-Stand test (number of repetitions) (38), Timed-up-and-go test (seconds) (39), maximal muscular strength (37) (kg, using a 10-repetition-maximum test for chest press, leg press, and horizontal seated row), and estimated relative VO_2max_ (Ebbling submaximal treadmill test) (40).

##### 2.3.2.5 Group allocation

The possible impact of the different study groups in BCS pain trajectories were taken into consideration by including dummy variables in the prediction models for each trial group (coded as 0: not included in the group; 1: being included in the group). To avoid multicollinearity, the exercise group was designated as the reference category against which the brief physical activity counseling and waitlist control groups were compared.

### 2.4 Statistical analysis

Statistical analyses were performed using SPSS® software (Version 29.0, Armonk, NY: IBM Corp) and R (Version 4.4.1). Following an intent-to-treat approach, missing data were handled using multivariate imputation by chained equations in R, utilizing a predictor matrix tailored to the study variables. Both pain and predictor variables were described using absolute (*n*) and relative (%) frequencies, or mean and standard deviation, depending on their nature (categorical or quantitative). Pain intensity tertiles at baseline and 4 months were categorized into three categories (low: scores from 0 to 3; medium: scores from 4 to 5; high: scores from 6 to 10). Pain change scores on pain intensity levels were categorized into three categories (less: reduction of pain intensity; similar: no significant changes in pain intensity; more: increase in pain intensity).

Multiple regression analyses were conducted to identify predictors of pain intensity at baseline, 4-month follow-up, and for change scores. To ensure comparability across the three models and maintain a consistent theoretical framework, a uniform set of 10 predictors was selected *a priori* based on the biopsychosocial model and previous literature. These variables were organized into five domains: 1) sociodemographic (relationship status, education level); 2) treatment-related (mastectomy, chemotherapy); 3) health-related (sleep quality); 4) physical function (Timed Up-and-Go, Sit-to-Stand, chest press strength); and 5) group allocation. Given the sample size (*N* = 110), we acknowledge the risk of overfitting and the limited statistical power when including numerous predictors. To mitigate this, the 10 variables were chosen as a restrained representation of the biopsychosocial framework. To ensure model robustness, multicollinearity was assessed using the variance inflation factor, with all values remaining below 3. Residual diagnostics, including visual inspection of P-P plots and scatterplots of standardized residuals, confirmed that assumptions of normality, linearity, and homoscedasticity were met.

To evaluate additional exploratory variables that could not be included in the regression models due to power constraints, a complementary analysis was performed. Comparisons between pain tertiles were conducted to test whether there were significant differences in predictor variables. Chi-square tests of independence were performed for categorical predictors. For continuous predictor variables, one-way ANOVAs with Bonferroni *post-hoc* comparisons were conducted. The level of significance was set at *p* ≤ 0.05 for all analyses.

## 3 Results

### 3.1 Sample description

**Figure 1:**
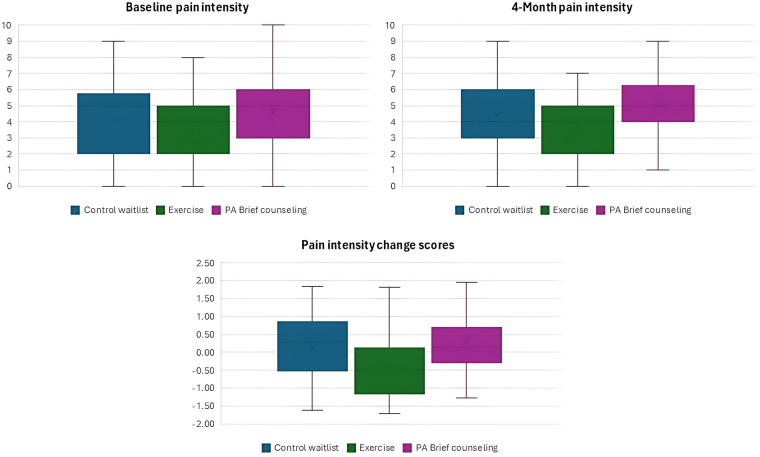
Pain intensity by intervention group.

A total of 110 participants (mean age 56.2 ± 7.7 years) reported a pain intensity of 4.2 (±2.3) and 4.4 (±2.2) at baseline and 4 months, respectively. Descriptives for pain intensity, socio-demographic, treatment-related, health-related, and physical function variables are presented in Table 1. Most participants (66.4%) were diagnosed with stage II breast cancer and had been receiving hormonal therapy for an average of 22 months. The most common treatment procedures included mastectomy (75.5%), chemotherapy (70.0%), and radiotherapy (82.7%). The baseline, 4-month, and change scores pain intensity differences across intervention groups are represented in Figure 1.

**Table 1:** Descriptives for pain intensity, pain tertiles, socio-demographic, treatment-related, health-related, and physical function variables are presented.

Variables	*n*	%	Variables	Mean ± SE	Min-Max
Baseline Pain Tertiles			**Pain**		
Low	41	37,3	Baseline Pain Intensity	4.2 ± 2.3	0–10
Medium	40	36,4	4-Month Pain Intensity	4.4 ± 2.2	0–9
High	20	26,4	**Socio-Demographic**		
4-Month Pain Tertiles			Age	56.2 ± 7.7	25–69
Low	35	31,8	**Treatment-related**		
Medium	43	38,2	Time Since Diagnosis	34.7 ± 21.6	4–105
High	29	30,0	Time on Hormonal Therapy	22.1 ± 17.6	1–74
Relationship status			**Health-related**		
Being single	41	37.3	Depression (HADS)	9.8 ± 3.2	7–24
Having a partner	69	62.7	Sleep Time	6.7 ± 0.9	4.3–9.4
Education level			Sleep Quality (PSQI)	2.6 ± 0.8	1–4
Elementary school	16	14.5	Waist Circumference	89.7 ± 11.6	63.5–124.4
Middle School	19	17.3	Body Fat Percentage	36.5 ± 5.4	18.3–47.7
High School	19	17.3	**Physical Function**		
Bachelor's Degree	41	37.3	Handgrip	20.0 ± 5.8	0–32
Master's Degree or Higher	15	13.6	Timed up-and-go	5.0 ± 0.8	3.2–7.5
Financial situation perception			Sit to Stand	14.1 ± 4.0	7–25
Difficult	15	13.6	Chest Press Strength	11.9 ± 4.0	0–20
Enough	54	49.1	Leg Press Strength	77.1 ± 24.6	20–140
Comfortable	41	37.3	Horizontal Row Strength	19.2 ± 4.9	5–27.3
Cancer Stage			VO2max	29.8 ± 5.1	18.6–42.1
Stage I	16	14.5			
Stage II	73	66.4			
Stage III	21	19.1			
Mastectomy					
No	27	24.5			
Yes	83	75.5			
Axillary Lymph Node Dissection					
No	45	40.9			
Yes	65	59.1			
Expander Placement					
No	98	89.1			
Yes	12	10.9			
Chemotherapy					
No	33	30.0			
Yes	77	70.0			
Radiotherapy					
No	19	17.3			
Yes	91	82.7			
Lumpectomy					
No	91	82.7			
Yes	19	17.3			
Chronic diseases					
No	44	40.0			
Yes	66	60.0			
Group Allocation					
Exercise Group	40	36.4			
Brief Counseling Group	36	32.7			
Waitlist Control Group	34	30.9			

### 3.2 Predictors of baseline, 4-month, and change scores of pain intensity

Table 2 presents bivariate analyses between potential predictors and average pain intensity at baseline, 4-month, and change scores. At baseline, significant positive associations were observed with having a partner (*r* = 0.23, *p* < 0.05), waist circumference (*r* = 0.21, *p* < 0.05), body fat percentage (*r* = 0.24, *p* < 0.05) and timed-up-and-go (*r* = 0.28, *p* < 0.01), and negative associations with higher educational level (*r* = −0.35, *p* < 0.001), sleep time (*r* = −0.19, *p* < 0.05), sleep quality (*r* = −0.37, *p* < 0.001), handgrip strength (*r* = −0.19, *p* < 0.05), and sit-to-stand (*r* = −0.20, *p* < 0.05). The 4-month correlations showed significant and positive associations with expander placement (*r* = 0.19, *p* < 0.05), body fat percentage (*r* = 0.23, *p* < 0.05) and timed up-and-go (*r* = 0.23, *p* < 0.05), and negative associations with a higher educational level (*r* = −0.28, *p* < 0.01), sleep quality (*r* = −0.20, *p* < 0.05), handgrip strength (*r* = −0.28, *p* < 0.01), sit-to-stand (*r* = −0.27, *p* < 0.01), and chest press strength (*r* = −0.31, *p* < 0.001). Change scores for average pain correlations' demonstrated significant and positive associations with having a partner (*r* = −0.22, *p* < 0.05), and negative associations with handgrip strength (*r* = −0.20, *p* < 0.05), sit-to-stand (*r* = −0.19, *p* < 0.05), chest press strength (*r* = −0.25, *p* < 0.01), horizontal row strength (*r* = −0.19, *p* < 0.05), and VO2max (*r* = −0.22, *p* < 0.05).

**Table 2:** Bivariate correlations between potential predictors and average pain intensity at baseline, 4-month, and change scores.

Variables	Average Pain Intensity		
	Baseline	4-Month	Change Scores
Socio-Demographic			
Age	−0.17	−0.10	0.13
Having a partner	0.23*	−0.02	−0.22*
Higher Educational Level	−0.35***	−0.28**	−0.08
Better perception of the Financial Situation	−0.16	−0.12	−0.02
Treatment-related			
Breast Cancer Stage	0.07	0.09	0.06
Time Since Diagnosis	−0.13	−0.09	−0.10
Time on Hormonal Therapy	−0.14	−0.11	−0.03
Mastectomy	0.05	−0.03	−0.08
Axillary Lymph Node Dissection	0.11	0.09	0.03
Expander Placement	0.12	0.19*	0.15
Chemotherapy	0.13	0.18	0.12
Radiotherapy	0.17	0.00	−0.14
Lumpectomy	−0.08	−0.02	0.04
Health-related			
Chronic Diseases	0.03	0.02	0.00
Depression (HADS)	0.17	0.07	−0.05
Sleep Time	−0.19*	−0.16	−0.05
Sleep Quality (PSQI)	−0.37***	−0.20*	0.04
Waist Circumference	0.21*	0.17	0.05
Body Fat Percentage	0.24*	0.23*	0.11
Physical Function			
Handgrip	−0.19*	−0.28**	−0.20*
Timed up-and-go	0.28**	0.23*	0.07
Sit to Stand	−0.20*	−0.27**	−0.19*
Chest Press Strength	−0.18	−0.31***	−0.25**
Leg Press Strength	0.04	−0.11	−0.17
Horizontal Row Strength	−0.02	−0.16	−0.19*
VO2max	0.10	−0.16	−0.22*

* *p* < .05.

** *p* < .01.

*** *p* < .001.

Multiple regression analyses were conducted to identify predictors of pain intensity using a biopsychosocial framework. Predictive models for baseline, 4-month, and change scores for pain intensity are presented in Tables 3–5, respectively. For all longitudinal models, the exercise group was utilized as the reference category for the intervention domain.

**Table 3:** Predictors of baseline pain intensity.

	Model 1			
	*β* (SE)	(95% CI)	*p*	VE (%)
Constant	5.524 (2.367)	(0.827, 10.221)	**0**.**022**	
Sociodemographic variables				
Having a Partner	1.044 (0.415)	(0.220, 1.868)	**0**.**014**	**1**.**275**
Higher Educational Level	−0.209 (0.177)	(−0.560, 0.143)	0.242	0.357
Treatment-related variables				
Mastectomy	0.285 (0.468)	(−0.643, 1.214)	0.544	0.075
Chemotherapy	0.201 (0.422)	(−0.636, 1.038)	0.635	0.043
Health-related variables				
Sleep Quality (PSQI)	−1.154 (0.272)	(−1.694, −0.615)	**0**.**000**	**3**.**727**
Physical Function variables				
Timed up-and-go	0.593 (0.298)	(0.002, 1.185)	**0**.**049**	**1**.**107**
Sit to Stand	−0.031 (0.054)	(−0.137, 0.076)	0.571	0.076
Chest Press Strength	−0.102 (0.052)	(−0.205, 0.001)	0.053	0.834
Intervention Groups				
Brief Physical Activity Counseling	0.316 (0.479)	(−0.634, 1.267)	0.510	0.110
Waitlist Control	−0.212 (0.483)	(−1.171, 0.746)	0.661	0.048
F	5.072			
*Degrees of freedom*	10			
R^2^	0.339			
R^2^_adjust_	0.272			
*P*	**<0.001**			

VE, variance explained (proportion of explained variance in the dependent variable attributed to each of the considered predictors).

**Table 4:** Predictors of 4-month pain intensity .

	Model 2			
	β (SE)	(95% CI)	*p*	VE (%)
Constant	6.779 (2.281)	(2.253, 11.306)	**0**.**004**	
Sociodemographic variables				
Having a Partner	−0.132 (0.400)	(−0.926, 0.662)	0.743	0.018
Higher Educational Level	−0.232 (0.171)	(−0.571, 0.106)	0.177	0.401
Treatment-related variables				
Mastectomy	−0.152 (0.451)	(−1.047, 0.743)	0.737	0.019
Chemotherapy	0.307 (0.407)	(−0.499, 1.114)	0.451	0.092
Health-related variables				
Sleep Quality (PSQI)	−0.574 (0.262)	(−1.095, −0.054)	**0**.**031**	0.836
Physical Function variables				
Timed up-and-go	0.301 (0.287)	(−0.269, 0.871)	0.297	0.258
Sit to Stand	−0.050 (0.052)	(−0.153, 0.052)	0.334	0.185
Chest Press Strength	−0.131 (0.050)	(−0.231, −0.032)	**0**.**010**	**1**.**255**
Intervention Groups				
Brief Physical Activity Counseling	1.240 (0.461)	(0.324, 2.155)	**0**.**008**	**1**.**535**
Waitlist Control	0.719 (0.466)	(−0.205, 1.643)	0.126	0.501
F	3.921			
*Degrees of freedom*	10			
R^2^	0.284			
R^2^_adjust_	0.211			
*p*	**<0.001**			

VE, variance explained (proportion of explained variance in the dependent variable attributed to each of the considered predictors).

**Table 5:** Predictors of pain intensity change scores.

	Model 3			
	β (SE)	(95% CI)	*p*	VE (%)
Constant	0.941 (1.103)	(−1.247, 3.130)	0.395	
Sociodemographic variables				
Having a Partner	−0.441 (0.194)	(−0.825, −0.057)	**0**.**025**	0.580
Higher Educational Level	−0.065 (0.082)	(−0.229, 0.099)	0.431	0.089
Treatment-related variables				
Mastectomy	−0.189 (0.218)	(−0.622, 0.243)	0.387	0.085
Chemotherapy	0.112 (0.197)	(−0.278, 0.502)	0.569	0.034
Health-related variables				
Sleep Quality (PSQI)	0.061 (0.127)	(−0.191, 0.312)	0.633	0.026
Physical Function variables				
Timed up-and-go	−0.028 (0.139)	(−0.303, 0.248)	0.842	0.006
Sit to Stand	−0.019 (0.025)	(−0.069, 0.030)	0.445	0.076
Chest Press Strength	−0.042 (0.024)	(−0.091, 0.006)	0.083	0.369
Intervention Groups				
Brief Physical Activity Counseling	0.625 (0.223)	(0.183, 1.068)	**0**.**006**	**1**.**098**
Waitlist Control	0.500 (0.225)	(0.054, 0.947)	**0**.**029**	0.682
F	2.560			
*Degrees of freedom*	10			
R^2^	0.205			
R^2^_adjust_	0.125			
*p*	**0**.**009**			

VE, variance explained (proportion of explained variance in the dependent variable attributed to each of the considered predictors.

The prediction model for baseline pain intensity (model 1) was statistically significant, accounting for 27.2% of the variance (*R^2^adj* = 0.272, *p* < 0.001). Having a partner (95% CI: 0.220, 1.868, *p* = 0.014), lower sleep quality (95% CI: −1.694, −0.615, *p* < 0.000), and additional time on the Timed-up-and-go test (95% CI: 0.002, 1.185, *p* = 0.049) were significant predictors of higher baseline pain intensity. While not statistically significant, lower chest press strength (95% CI: −0.205, 0.001, *p* = 0.053) approached significance.

Regarding the 4-month pain intensity (model 2), the prediction model explained 21.1% of the variance (*R^2^adj* = 0.211, *p* < 0.001). Significant predictors of higher 4-month pain included lower sleep quality (95% CI: −1.095, −0.054, *p* = 0.031), and being in the brief physical activity counseling group (95% CI: 0.324, 2.155, *p* = 0.008) relative to the exercise group. Conversely, higher chest press strength (95% CI: −0.231, −0.032, *p* = 0.010) was a significant predictor of lower pain intensity at the 4-month follow-up.

Considering pain intensity change scores (model 3), the model accounted for 12.5% of the variance (*R^2^adj* = 0.125, *p* < 0.009). Compared to the exercise group, being in the brief physical activity counseling group (95% CI: 0.183, 1.068, *p* = 0.006), and waitlist control group (95% CI: 0.054, 0.947, *p* = 0.029) significantly predicted an increase in pain intensity over time. Conversely, having a partner (95% CI: −0.825, −0.057, *p* = 0.025 significantly predicted a reduction in pain intensity.

### 3.3 Comparisons amongst pain tertiles

**Figure 2:**
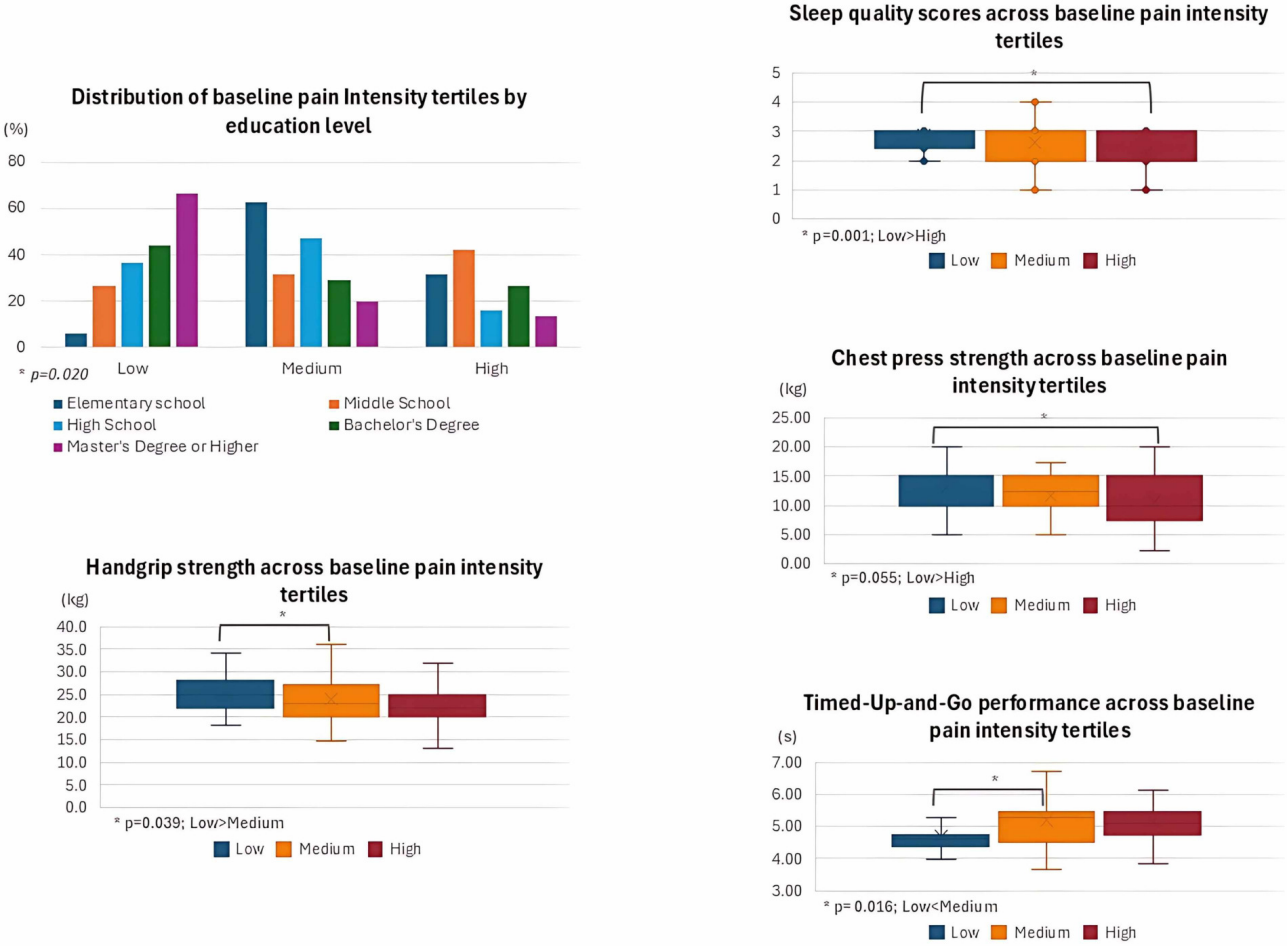
Significant results for the baseline tertile comparison.

**Figure 3:**
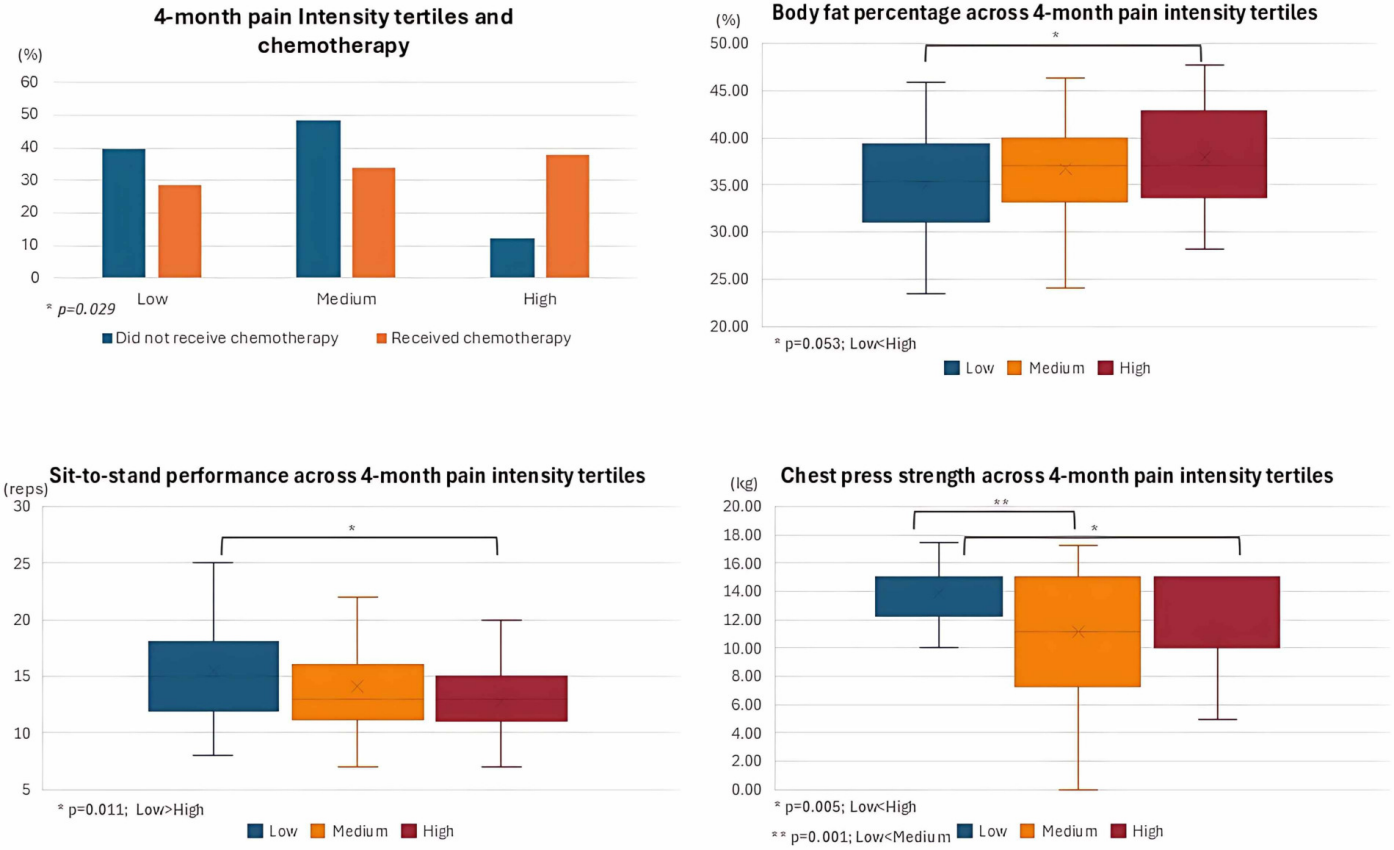
Significant results for the 4-month tertile comparison.

Comparisons amongst pain tertiles for baseline, 4-month, and change scores for categorical and continuous predictors are presented in Tables 6, 7, respectively. For visual clarity, graphical representation of significant results for baseline and 4-month tertile comparison are presented in Figures 2, 3, respectively.

**Table 6:** Tertile comparison for categorical predictors.

Variables	Frequency of baseline pain levels %			*χ* ^2^	Cramer’ V	*p-value*	Frequency of 4-Month pain levels %			*χ* ^2^	Cramer's V	*p-value*	Frequency of Pain Change Scores levels %			*χ* ^2^	Cramer's V	*p-value*
	Low	Medium	High				Low	Medium	High				Less	Similar	More			
**Total**	37.3	36.4	26.4				31.8	38.2	30.0				33.6	35.5	30.9			
Relationship status																		
Being single	51.2	29.3	19.5	5.480	0.2	0.069	29.3	39.0	31.7	0.209	0.0	0.909	22.0	39.0	39.0	4.281	0.2	0.114
Having a partner	29.0	40.6	30.4				33.3	37.7	29.0				40.6	33.3	26.1			
Education level																		
Elementary school	6.3	62.5	31.3	17.841	**0**.**3**	**0**.**020**^a^	12.5	37.5	50.0	7.981	0.2	0.447^a^	18.8	50.0	31.3	3.681	0.1	0.890^a^
Middle School	26.3	31.6	42.1				26.3	42.1	31.6				31.6	31.6	36.8			
High School	36.8	47.4	15.8				26.3	42.1	31.6				36.8	26.3	36.8			
Bachelor's Degree	43.9	29.3	26.8				39.0	34.1	26.8				36.6	36.6	26.8			
Master's Degree or Higher	66.7	20.0	13.3				46.7	40.0	13.3				40.0	33.3	26.7			
Financial situation perception																		
Difficult	26.7	26.7	46.7	4.483	0.1	0.400^a^	26.7	26.7	46.7	3.402	0.1	0.518^a^	33.3	13.3	53.3	8.180	0.2	0.090^a^
Enough	35.2	40.7	24.1				31.5	37.0	31.5				29.6	46.3	24.1			
Comfortable	43.9	34.1	22.0				34.1	43.9	22.0				39.0	29.3	31.7			
Cancer Stage																		
Stage I	43.8	50.0	6.3	4.237	0.1	0.333^a^	43.8	37.5	18.8	2.751	0.1	0.609^a^	37.5	43.8	18.8	8.391	0.2	0.077^a^
Stage II	37.0	32.9	30.1				28.8	41.1	30.1				38.4	27.4	34.2			
Stage III	33.3	38.1	28.6				33.3	28.6	38.1				14.3	57.1	28.6			
Mastectomy																		
No	40.7	40.7	18.5	1.139	0.1	0.570	33.3	29.6	37.0	1.302	0.1	0.549	29.6	22.2	48.1	5.378	0.2	0.066
Yes	36.1	34.9	28.9				31.3	41.0	27.7				34.9	39.8	25.3			
Axillary Lymph Node Dissection																		
No	42.2	26.7	31.1	3.121	0.2	0.223	31.1	46.7	22.2	2.983	0.2	0.225	35.6	35.6	28.9	0.184	0.0	0.941
Yes	33.8	43.1	23.1				32.3	32.3	35.4				32.3	35.4	32.3			
Expander Placement																		
No	39.8	35.7	24.5	2.835	0.2	0.234^a^	34.7	37.8	27.6	4.174	0.2	0.113^a^	35.7	34.7	29.6	1.804	0.1	0.409^a^
Yes	16.7	41.7	41.7				8.3	41.7	50.0				16.7	41.7	41.7			
Chemotherapy																		
No	42.4	36.4	21.2	0.810	0.2	0.700	39.4	48.5	12.1	7.184	**0**.**3**	**0**.**029**	42.4	36.4	21.2	2.528	0.2	0.299
Yes	35.1	36.4	28.6				28.6	33.8	37.7				29.9	35.1	35.1			
Radiotherapy																		
No	47.4	42.1	10.5	3.021	0.2	0.205^a^	31.6	42.1	26.3	0.197	0.0	0.950	21.1	47.4	31.6	2.004	0.1	0.395^a^
Yes	35.2	35.2	29.7				31.9	37.4	30.8				36.3	33.0	30.8			
Lumpectomy																		
No	36.3	36.3	27.5	0.391	0.1	0.861	30.8	39.6	29.7	0.464	0.1	0.814	34.1	38.5	27.5	3.386	0.2	0.198^a^
Yes	42.1	36.8	21.1				36.8	31.6	31.6				31.6	21.1	47.4			
Chronic diseases																		
No	34.1	34.1	31.8	0.521	0.1	0.804	34.1	34.1	31.8	0.521	0.1	0.804	36.4	31.8	31.8	0.455	0.1	0.831
Yes	30.3	40.9	28.8				30.3	40.9	28.8				31.8	37.9	30.3			

a Fisher's Exact Test (number of cases lower than 5) | Cramer's V association effect size—Small effect: 0.1; Medium effect: 0.3; Large effect: 0.5.

**Table 7:** Tertile comparison for continuous predictors.

Variables	Baseline Pain Intensity Levels						Post Hoc Test	4-Month Pain Intensity Levels						Post Hoc Test	Pain Change Scores of Pain Intensity Levels						Post Hoc Test
	Low		Medium		High			Low		Medium		High			Less		Similar		More		
	*N* = 41		*N* = 40		*N* = 29			*N* = 35		*N* = 42		*N* = 33			*N* = 37		*N* = 39		*N* = 34		
	Mean ± SE	95% CI	Mean ± SE	95% CI	Mean ± SE	95% CI		Mean ± SE	95% CI	Mean ± SE	95% CI	Mean ± SE	95% CI		Mean ± SE	95% CI	Mean ± SE	95% CI	Mean ± SE	95% CI	
Age	56.6 ± 1.0	54.5; 58.7	57.8 ± 1.4	55.0; 60.6	53.5 ± 1.4	50.7; 56.4		55.7 ± 1.1	53.4; 57.9	56.4 ± 1.3	53.7; 59.0	56.6 ± 1.4	53.8; 59.4		54.7 ± 1.2	52.4; 57.1	56.7 ± 1.3	54.1; 59.4	57.2 ± 1.3	54.5; 60	
Time Since Diagnosis	38.9 ± 3.6	31.6; 46.1	32.1 ± 3.3	25.4; 38.8	32.4 ± 3.8	24.6; 40.3		38.1 ± 3.6	30.9; 45.3	33.3 ± 3.3	26.7; 39.9	33.0 ± 4.0	24.8; 41.1		35.6 ± 3.2	29; 42.2	37.7 ± 4	29.7; 45.7	30.3 ± 3.4	23.5; 37.2	
Time on Hormonal Therapy	25.9 ± 3.0	19.9; 31.9	18.5 ± 2.5	13.5; 23.4	21.6 ± 3.3	15.0; 28.3		24.8 ± 3.0	18.8; 30.9	21.5 ± 2.4	16.7; 26.3	19.8 ± 3.5	12.6; 27.0		22.9 ± 2.6	17.8; 28.1	24.7 ± 3	18.5; 30.8	18.1 ± 3.1	11.9; 24.4	
Depression (HADS)	9.3 ± 0.5	8.2; 10.3	9.8 ± 0.4	8.9; 10.6	10.7 ± 0.7	9.3; 12.0		9.6 ± 0.5	8.5; 10.6	9.6 ± 0.5	8.7; 10.6	10.3 ± 0.6	9.1; 11.6		10 ± 0.5	9; 11	10 ± 0.5	9; 11.1	9.4 ± 0.6	8.2; 10.6	
Sleep Time	6.9 ± 0.1	6.6; 7.2	6.6 ± 0.2	6.2; 6.9	6.4 ± 0.1	6.2; 6.7		6.8 ± 0.2	6.5; 7.2	6.7 ± 0.1	6.4; 7.0	6.4 ± 0.1	6.1; 6.7		6.7 ± 0.2	6.3; 7	6.8 ± 0.1	6.5; 7.1	6.5 ± 0.1	6.2; 6.8	
Sleep Quality (PSQI)	2.9 ± 0.1	2.7; 3.1	2.6 ± 0.1	2.4; 2.8	2.3 ± 0.1	2.0; 2.6	**Low** **>** **High**	2.7 ± 0.1	2.5; 3.0	2.7 ± 0.1	2.5; 2.9	2.4 ± 0.1	2.2; 2.7		2.6 ± 0.1	2.3; 2.8	2.7 ± 0.1	2.4; 2.9	2.6 ± 0.1	2.4; 2.9	
Waist Circumference	87.5 ± 1.9	83.6; 91.4	91.1 ± 1.6	87.9; 94.4	90.9 ± 2.3	86.3; 95.6		87.7 ± 2.1	83.4; 92.0	90.0 ± 1.7	86.6; 93.4	91.6 ± 2.0	87.5; 95.6		88 ± 1.9	84.3; 91.8	91.7 ± 2	87.6; 95.9	89.3 ± 1.8	85.6; 93	
Body Fat Percentage	35.5 ± 0.9	33.7; 37.3	36.6 ± 0.8	34.9; 38.2	37.9 ± 0.9	35.9; 39.8		34.9 ± 1.0	32.8; 37.0	36.7 ± 0.7	35.3; 38.1	38.0 ± 1.0	36.0; 39.9	**Low** **<** **High**^b^	35.4 ± 1	33.4; 37.4	37.2 ± 0.8	35.7; 38.8	36.9 ± 0.9	35.1; 38.7	
Handgrip	21.9 ± 0.7	20.6; 23.3	18.8 ± 1.1	16.5; 21.0	19.0 ± 0.9	17.1; 20.9	**Low** **>** **Medium**	21.5 ± 1.0	19.4; 23.5	20.1 ± 0.6	18.8; 21.4	18.3 ± 1.2	15.8; 20.8		21.1 ± 1	19.1; 23	20.7 ± 0.7	19.2; 22.2	18.0 ± 1.1	15.7; 20.3	
Timed up-and-go	4.7 ± 0.1	4.5; 4.9	5.2 ± 0.1	4.9; 5.4	5.1 ± 0.1	4.8; 5.4	**Low** **<** **Medium**	4.7 ± 0.1	4.5; 4.9	5.1 ± 0.1	4.8; 5.4	5.1 ± 0.2	4.8; 5.4		4.8 ± 0.1	4.6; 5	5 ± 0.1	4.7; 5.3	5.1 ± 0.2	4.7; 5.4	
Sit to Stand	15.1 ± 0.7	13.7; 16.5	13.4 ± 0.6	12.2; 14.6	13.7 ± 0.7	12.3; 15.2		15.5 ± 0.7	14.0; 17.0	14.1 ± 0.6	12.8; 15.3	12.7 ± 0.6	11.5; 13.9	**Low** **>** **High**	15.1 ± 0.6	13.8; 16.3	13.8 ± 0.7	12.4; 15.2	13.4 ± 0.6	12.1; 14.7	
Chest Press Strength	13.0 ± 0.5	11.9; 14.1	11.5 ± 0.6	10.3; 12.8	10.7 ± 0.8	9.0; 12.4	**Low** **>** **High**^a^	13.9 ± 0.5	12.9; 15.0	11.2 ± 0.6	9.9; 12.4	10.5 ± 0.7	9.1; 11.9	**Low** **>** **High; Low** **>** **Medium**	13.2 ± 0.6	11.9; 14.4	11.3 ± 0.7	9.9; 12.7	11.0 ± 0.6	9.7; 12.3	**ND**
Leg Press Strength	73.2 ± 2.9	67.4; 79.0	85.1 ± 3.9	77.2; 93.0	71.6 ± 5.5	60.3; 83.0	**ND**	77.8 ± 3.6	70.5; 85.0	82.5 ± 3.9	74.6; 90.4	69.4 ± 4.5	60.2; 78.6		79.4 ± 3.9	71.5; 87.3	78.7 ± 4.2	70.3; 87.1	72.7 ± 4.2	64.2; 81.2	
Horizontal Row Strength	19.1 ± 0.7	17.7; 20.5	20.1 ± 0.8	18.4; 21.8	18.0 ± 0.9	16.1; 20.0		20.1 ± 0.6	18.8; 21.4	19.6 ± 0.9	17.8; 21.4	17.7 ± 0.9	15.9; 19.4		19.6 ± 0.8	18.1; 21.2	20.2 ± 0.7	18.7; 21.7	17.5 ± 0.9	15.7; 19.4	
VO2max	30.4 ± 0.7	28.9; 32.0	29.4 ± 0.8	27.8; 31.1	29.4 ± 1.0	27.3; 31.5		30.9 ± 0.9	29.1; 32.7	29.9 ± 0.8	28.3; 31.5	28.4 ± 0.9	26.7; 30.2		31.4 ± 0.8	29.7; 33.1	28.9 ± 0.9	27.1; 30.7	29.1 ± 0.7	27.6; 30.6	

a *p* = 0.055.

b *p* = 0.053.

ND = no differences between pain intensity tertiles.

At baseline, 37.3% of participants reported low pain intensity, 36.4% reported medium pain, and 26.4% reported high pain. Higher education levels were different amongst tertiles, with lower pain intensity (elementary school = 6.3%, middle school = 26.3%, high school = 36.8%, bachelor's degree = 43.9%, and master's degree or higher = 66.7%; *p* = 0.020). Participants with lower pain levels reported better sleep quality compared to those with higher pain levels [MD [95% CI]: 0.6 [2.9; 2.3], *p* = 0.001]. For both handgrip [MD [95% CI]: 3.1 [21.9; 18.8], *p* = 0.039] and timed up-and-go [MD [95% CI]: −0.5 [4.7; 5.2], *p* = 0.016], the low pain intensity group performed better than the medium pain intensity group. Chest press strength only revealed a marginally significant difference between tertiles, and *post-hoc* comparisons did not reveal significant differences between low and high pain levels [MD [95% CI]: 2.3 [13.0; 10.7], *p* = 0.055]. Slight significant differences between groups were also reported for leg press strength, though the *post-hoc* test did not reveal significant differences between medium and high pain levels [MD [95% CI]: 13.5 [85.1; 71.6], *p* = 0.072].

The relative frequency for 4-month pain intensity was 31.8, 38.2, and 30.0% for low, medium, and high pain tertile groups, respectively. Receiving chemotherapy was different amongst tertiles, with higher pain intensity (low pain = 12.1% vs. high pain = 37.7%, *p* = 0.029). The 4-month comparisons for the sit-to-stand test [MD [95% CI]: 2.8 [15.5; 12.7], *p* = 0.011] indicated significant differences between low and high pain levels, with higher performance status being observed in participants with low pain levels. Participants with low pain levels exhibited significantly higher chest press strength compared to those with medium [MD [95% CI]: 2.7 [13.9; 11.2], *p* = 0.005] or high [MD [95% CI]: 3.4 [13.9; 10.5], *p* = 0.001] pain levels. Borderline significant differences in body fat percentage were observed between tertiles, but *post-hoc* analysis revealed no significant differences between low and high pain levels [MD [95% CI]: −3.1 [34.9; 38.0], *p* = 0.053].

Regarding pain intensity change scores, 34.6% revealed less pain, 35.5% similar pain, and 30.9% more pain from baseline to 4 months. There were no significant differences among change-score pain tertiles and categorical variables. Concerning continuous predictors, comparisons between pain tertiles revealed slightly significant differences in chest press strength; however, *post-hoc* analysis indicated no significant differences between less and more pain change-score levels [MD [95% CI]: 2.2 [13.2; 11.0], *p* = 0.061].

## 4 Discussion

This study investigated baseline predictors of pain intensity in BCS undergoing AIs through a longitudinal analysis of the PAC-WOMAN trial. Predictors were categorized into five domains: sociodemographic, treatment-related, health-related, physical function, and intervention groups. In addition, these factors were analyzed across different pain intensity tertiles.

The baseline pain intensity prediction model explained 27.2% of the variance, representing a medium-to-large effect size (41). Model 1 revealed having a partner, lower sleep quality, and lower chest press strength as significant predictors of higher baseline pain intensity. The association between having a partner and higher pain levels contrasts with previous studies (42, 43) and, given that our assessment of relationship status was limited to a binary variable, these findings should be interpreted with caution. The observed association may be influenced by unmeasured confounding factors, such as the quality of social support or the specific division of domestic labor not captured by binary relationship status. Concerning health-related variables, poor sleep quality was strongly associated with higher baseline pain intensity. This aligns with the established bidirectional relationship between sleep and pain (44, 45), where poor sleep quality increases pain sensitivity, while pain is one of the most reported factors underlying poor sleep quality. Furthermore, additional time on the timed-up-and-go test was significantly associated with higher baseline intensity pain. This observation is consistent with the current literature, suggesting that improved physical function can mitigate pain intensity among BCS (32).

Regarding model 2, the prediction analysis for pain intensity at 4-month revealed a medium effect size with 21.1% of the explained variance (41). Over time, significant predictors shifted; while sleep quality remained a significant health-related factor, lower chest press strength emerged as a relevant predictor. This suggests that upper-body muscular strength may serve as a protective factor against the progression of pain in BCS undergoing AI therapy. Higher levels of muscular strength are often associated with better joint stability, which may mitigate the musculoskeletal side effects commonly associated with AIs (46). Furthermore, chest press strength is a proxy for functional capacity; survivors with greater upper-body strength may engage more easily in daily activities, preventing the cycle of “pain-related kinesiophobia” and subsequent physical deconditioning that often exacerbates the pain experience in this population (47). Furthermore, the intervention domain became relevant. Compared to the exercise group, participants receiving only brief physical activity counseling reported higher pain levels. This finding may suggest that physical activity educational interventions may be insufficient to mitigate pain over time, emphasizing the need for supervised exercise prescription to achieve meaningful clinical outcomes (48).

The final predictive model for pain intensity change scores accounted for 12.5% of the variance, indicating a small-to-medium effect size. In model 3, both the physical activity counseling and waitlist control groups were associated with an increase in pain intensity relative to the supervised exercise group. These results reinforce the possible protective role of a supervised exercise prescription setting in mitigating pain intensity levels amongst BCS. Additionally, having a partner was significantly associated with a reduction in pain intensity over the intervention period. Although having a partner was linked to higher pain at baseline, the direction of this association reversed over time. The shift in the direction of this association over time highlights the need for multidimensional measures of social support, caregiving load, and relationship dynamics to clarify the role of social structures in BCS pain trajectories.

Notably, the proportion of explained variance decreased from baseline (30.3%) to 4 months (23.3%) and change scores (14.4%). This decline suggests that while baseline characteristics strongly influence BCS's pain experience before participating in PA interventions, they may not be the primary drivers of persistent pain over time or changes in pain levels. Pain trajectories appear to be highly individualized and influenced by biopsychosocial factors, thus, challenging to predict using baseline variables. Perhaps unmeasured variables, such as psychosocial stress, coping strategies, or the impact of ongoing aromatase inhibitor therapy, may provide valuable insights into individual pain management, specifically for PA interventions.

Exploratory analysis using comparisons across pain intensity tertiles provided additional insights into the variables associated with pain intensity. These results only revealed significant differences amongst pain intensity tertiles at both baseline and 4-month. At baseline, participants in the lower pain intensity tertile were more likely to have higher education levels, better sleep quality, greater handgrip strength, and better performance on the timed up-and-go test. At 4 months, chemotherapy was associated with the higher pain intensity tertile, while better sit-to-stand performance and greater chest press strength were associated with the lower pain intensity tertile.

For sociodemographic characteristics, treatment-related factors, and health-related variables, comparisons were not consistent between baseline and 4-month pain intensity. Lower education levels, which might be associated with lower health literacy, impacting the understanding of cancer-related pain and possible management strategies (34, 42, 43), and poor sleep quality were only associated with the lower pain intensity tertile at baseline. These findings reinforced the importance of interventions targeting health literacy programs, particularly for those with lower educational levels, and the relevance of monitoring sleep routine, disturbances and integrating possible sleep improvement techniques. Conversely, receiving chemotherapy, a treatment-related pain predictor often mentioned in the literature (19), was only associated with higher 4-month pain intensity.

In contrast, for the physical function domain, this analysis emphasized the strong association between better physical function and low pain intensity for both baseline and 4-month. These findings suggest that better physical fitness and integrating strength and functional mobility testing and exercising into rehabilitation programs may act as protective factors against higher pain levels for BCS.

Some limitations of this study should be acknowledged to contextualize the findings and guide future research. Participants age cutoff of under 70 and their motivation to engage in a PA intervention, potentially introduced selection bias, which would limit the representativeness of this sample regarding the relationships between predictors and pain outcomes. While our models were grounded in a biopsychosocial framework, the sample size of 110 participants relative to the number of predictors may limit the generalizability of the findings. These results should be considered exploratory and serve as a foundation for larger confirmatory trials. Finally, the study primarily focused on pain intensity on average; other pain multidimensional outcomes (e.g., interference, temporal patterns) could have added further exploration of pain experiences among BCS.

### 4.1 Practical implications and future directions

The present study provides valuable insights into the predictors of pain intensity in BCS on AIs, particularly within the context of PA interventions. By exploring different biopsychosocial domains for possible baseline predictors, this study contributes to a deeper understanding of the complex interplay of factors influencing pain persistence (or resolution) among this population. Furthermore, the prospective analyses, examining changes in pain intensity over time, provide valuable information on the dynamic behavior of pain. These results demonstrate that some baseline characteristics, while associated with initial pain levels, may not consistently predict subsequent pain experiences.

Future studies should increase the sample size and broaden the type of breast cancer population. Hormonal therapy, specifically with AIs, can serve as a treatment-related predictor in further analyses. Also, the use of longitudinal designs should be pursued, implementing different methodologies such as mixed-effects modeling to capture repeated measures and time-varying effects more effectively. Adding other psychosocial behavior factors, biological markers, and dynamic variables and examining the temporal relationships between predictors and pain outcomes over time may add considerable value to the understanding of pain persistence or resolution.

## 5 Conclusion

This study aimed to investigate baseline predictors of pain intensity in BCS on AIs at different moments through the PAC-WOMAN Trial. Significant predictors changed across the time periods, with better sleep quality and exercise group membership emerging as the most consistent variables associated with lower pain intensity. These findings highlight the complex and multifaceted nature of pain in this population and the need for longitudinal studies with diverse populations, as well as advanced methodologies to capture the dynamic nature of pain and refine intervention strategies. The present work emphasizes the importance of addressing pain management from a multidimensional perspective. Tailored interventions targeting modifiable factors and incorporating psychosocial, biological, and lifestyle factors into a personalized approach to pain management will improve BCS's quality of life.

## Data Availability

The raw data supporting the conclusions of this article will be made available by the authors, without undue reservation.
